# Integrating Simulation-Based Education Into Regional Clinical Clerkship for Future Doctors: A Narrative Review

**DOI:** 10.7759/cureus.71903

**Published:** 2024-10-20

**Authors:** Nobuyasu Komasawa, Masanao Yokohira

**Affiliations:** 1 Community Medicine Education Promotion Office, Faculty of Medicine, Kagawa University, Miki-cho, JPN; 2 Department of Medical Education, Kagawa University, Miki-cho, JPN

**Keywords:** clinical clerkship, medical education, medical student, rural medicine, simulation

## Abstract

Regional healthcare is vital for providing accessible, community-oriented services, addressing local health needs, and reducing disparities between urban and rural areas. It also supports local society and economies by enhancing healthcare facilities and related jobs. However, rural medical education in Japan faces significant challenges. There is a disparity in healthcare resources and personnel between urban and rural areas, leading to shortages in rural regions. Retention of physicians in rural areas is low, necessitating environments that encourage long-term commitment. Tailored educational programs to meet specific regional needs and improved clinical training environments are crucial. Addressing these challenges will elevate healthcare standards and reduce disparities.

## Introduction and background

Regional healthcare plays a crucial role in several aspects. Firstly, it provides accessible and community-oriented medical services, ensuring that even remote areas have necessary healthcare within reach [1]. This accessibility is essential for early diagnosis and treatment of diseases, significantly improving health outcomes and reducing the burden on more centralized urban healthcare systems [2]. Secondly, it addresses region-specific healthcare needs, tailoring programs to fit local characteristics and demands. For example, rural areas might have higher incidences of certain conditions due to environmental or occupational factors, and tailored programs can better manage and prevent these conditions [3]. Moreover, enhancing regional healthcare can help reduce disparities between urban and rural areas, promoting equitable access to medical services. This equity is fundamental for social justice, ensuring that all individuals, regardless of their location, can achieve optimal health [4]. Additionally, by bolstering healthcare facilities and related jobs, regional healthcare contributes positively to the local economy. Healthier populations can work more effectively, and healthcare facilities often become significant local employers, stimulating economic growth. Overall, regional healthcare is essential for fostering community health and development, and creating resilient and sustainable communities [5].

However, there are several challenges in regional medical education. Firstly, there is a significant disparity in healthcare between urban and rural areas. Urban areas tend to have more advanced medical infrastructure and a higher concentration of healthcare professionals, while rural areas often face shortages of medical resources and personnel. This disparity necessitates significant improvements in regional medical education to ensure that rural areas are adequately served. Moreover, there is a low retention rate of physicians in rural areas. Many young doctors prefer to work in urban settings where they have more opportunities for career advancement and access to better facilities. Therefore, it is crucial to create environments that encourage young doctors to stay long-term in rural areas. This might include offering competitive salaries, providing opportunities for continuous professional development, and ensuring a good quality of life [6]. Additionally, addressing region-specific healthcare needs through tailored educational programs is crucial. These programs should be designed to equip healthcare professionals with the skills and knowledge necessary to address the unique health challenges of rural areas. The development of educational environments is also urgent, requiring support to enhance the quality of clinical training and internships. This might involve partnerships with local hospitals and clinics to provide hands-on experience and ensure that medical students are well-prepared to meet the demands of rural healthcare. Addressing these challenges is expected to elevate the overall standard of healthcare regionally and contribute to reducing healthcare disparities [7]. Improving regional medical education will not only benefit rural populations but also strengthen the entire healthcare system by producing well-rounded, adaptable healthcare professionals [8].

## Review

Redefining the role of student doctors in participatory clinical training

In Japan, despite the clinical training curriculum occupying a significant portion of the later years of medical school, most students traditionally began substantial medical practices only after obtaining their medical licenses during the initial clinical training period [9,10]. The conventional observational nature of pre-graduate clinical training posed a considerable challenge due to its significant deviation from early clinical training. The insufficient "legal basis" for medical actions by medical students during pre-graduate clinical training was considered a major contributing factor to the observation-only nature of these practices [11,12].

To address the need for seamless integration of pre- and post-graduate clinical education and clarify the legal standing of medical students' medical activities during clinical training, discussions were initiated, leading to amendments in medical laws. As of 2023, medical students who passed the nationwide common exam such as computer-based testing (CBT) and Objective Structured Clinical Examination (OSCE) in their fourth year are authorized to perform medical acts during clinical training under the supervision of a mentor, excluding prescription issuance [13,14]. Various medical education institutions are actively engaging in awareness campaigns, anticipating further seamless integration of pre- and post-graduate clinical education (Table 1).

**Table 1:** Overview of publicized medical acts by student doctors

Provisions on the Scope of Medical Acts	Each medical education institution's oversight department determines the scope of medical acts by Student Doctors. Simulation training is conducted for procedures with high physical or psychological invasiveness.
Caution and Excluded Medical Acts	Prescription issuance is excluded from medical acts. Creating diagnosis reports requires meticulous guidance.
Efforts for Patient Consent	[Initiatives to Deepen Understanding] Efforts are made to gain understanding of the publicization of medical acts through internal notices and public relations. [Patient Consent Matters] Comprehensive consent documents are obtained for inpatients. Individual consent efforts are made for invasive procedures. A dedicated contact point is established to address patient complaints.

From the learner's perspective, medical students typically conclude their participatory clinical training as "student doctors" in the later years of medical school. Following this phase, they proceed to obtain their medical licenses and officially transition into "clinical trainees." Therefore, the emphasis during this period is on acquiring a comprehensive set of skills, spanning the four critical years from the later stages of medical school to the initial clinical training period as licensed practitioners [15,16]. The participatory clinical training itself takes place not only in the controlled environment of university hospitals but also in various regional hospitals and clinics [17]. As a result, deliberate and conscious efforts towards the publicization and proper handling of medical acts conducted by student doctors are increasingly necessary, especially in the context of regional medical education, where the role of these students can have a more direct impact on local healthcare delivery.

Simulation-based education is regarded as highly effective in medical training, offering students the opportunity to practice medical procedures in a controlled, risk-free environment. However, it faces several challenges that need to be addressed to maximize its potential. One of the primary issues is the substantial funding required for the purchase, upkeep, and continuous maintenance of simulation equipment. Simulators and training devices come with a high price tag and ensuring that they are regularly updated with the latest software and kept in proper working order requires ongoing financial investment. Moreover, the success of simulation education is heavily dependent on the availability of skilled instructors who can guide students effectively through the simulated scenarios.

Unfortunately, the pool of instructors with the necessary expertise in simulation-based education is limited, making it difficult for many educational institutions to secure an adequate number of qualified personnel. This shortage poses a significant barrier, particularly as medical schools face increasing student enrollment. To accommodate the growing number of students, multiple learners often have to share a single simulation device, which limits the amount of individual hands-on practice time each student receives. This not only affects the quality of the training but also reduces opportunities for personalized feedback.

Addressing these multifaceted challenges requires collaboration across educational institutions, government agencies, and policymakers. It is essential to reassess the allocation of resources to ensure that simulation-based education is adequately supported. This might involve redesigning current educational systems, establishing sustainable funding models, investing in the development and training of more skilled instructors, and improving the overall educational environment. Such efforts will be crucial in ensuring that simulation-based education continues to be a viable and effective component of medical training in the future.

The potential of simulation-based education for publicized medical acts

Simulation-based education is widely utilized not only in healthcare but also in various other fields such as aviation and the legal sector. These industries rely on simulation to replicate complex, high-stakes environments where mistakes can have serious consequences, similar to the clinical setting in healthcare. Grounded in experiential learning theory, simulation-based education functions by enabling learners to conceptualize insights gained through practical experiences. This process mirrors real-world situations as learners engage in simulated scenarios that closely resemble clinical settings. Through this process, students not only gain experience but also undergo deep reflection on their actions, leading to a cycle of continuous improvement and the development of new approaches based on their previous experiences [18,19]. In clinical education, students typically acquire "experience" directly from patient interactions and real-life medical practice, while in simulation-based education, they engage with simulated environments that mimic these clinical situations. Both approaches emphasize a critical phase of reflective debriefing, where learners analyze their experiences in detail, extracting valuable lessons from both successes and mistakes. This reflective process is a shared element between the two educational methods, making simulation education a highly complementary component of traditional clinical education.

Simulation-based education has proven to be particularly effective in helping learners acquire both technical and non-technical skills necessary for performing invasive medical procedures. Technical skills refer to the hands-on, procedural abilities required for tasks like surgery, inserting catheters, or administering injections. On the other hand, non-technical skills, such as communication, teamwork, decision-making, and leadership, are equally critical in ensuring successful patient outcomes. Simulation education creates a safe environment in which students can practice both sets of skills without the risk of harming actual patients (Table 2) [20,21].

**Table 2:** Various simulation-based education method

Simulation strategy	Expected Skills	Example
Task Trainer	Technical Skill	Blood collection Intravenous Keep
Simulated Patient (Physical Examination)	Technical Skill	Physical Examination
Simulated Patient (Medical Interview)	Non-technical Skill	Medical Interview, Informed Consent
Scenario Training with Simulator	Technical Skill Non-technical Skill	Basic/Advanced Life Support Primary trauma care
Scenario Training with problem-based learning	Non-technical Skill	Community Collaboration Scenario Interprofessional Scenario
Virtual Reality/Augmented Reality	Non-technical Skill	Disaster Education

By incorporating real-world challenges in a controlled setting, students are able to refine their abilities under the guidance of skilled instructors, improving both their proficiency in medical procedures and their capacity to handle complex, high-pressure situations. The versatility of simulation education, combined with its ability to mimic the unpredictability and complexity of real-life clinical situations, makes it an indispensable tool for modern medical education. As a result, the integration of simulation-based training with clinical practice has become a critical aspect of preparing future healthcare professionals for the challenges they will face in real-world medical settings. Additionally, due to its high objectivity, simulation is utilized for comprehensive assessments. The widespread adoption of simulation-based education is attributed to its ability to acquire technical and non-technical skills for low-frequency events without endangering patients [22].

Considering factors such as invasiveness, difficulty, and embarrassment, each medical education institution is required to categorize medical acts performed by student doctors into "mandatory" and "recommended" items. For recommended items, foundational training in simulation education is anticipated, ensuring the quality of student doctors' medical acts.

The necessity of regional medical education to adapt to a data-driven society

Medical education is constantly evolving, shaped by advancements in the life sciences and shifts within the healthcare industry. The life sciences, particularly after the decoding of the human genome, have entered a new era of complexity. Comprehensive analyses of metabolomes, transcriptomes, and other molecular datasets have become possible, leading to new discoveries. This process has been further accelerated by the use of artificial intelligence (AI), which has enabled more sophisticated data analysis and decision-making. These developments are driving continuous and complex changes in both medical research and education [23]. Moreover, the clinical field is experiencing its own set of transformations. Trends such as the "coexistence of specialization and comprehensive care," as well as "shifts in access to medical knowledge and growing awareness of patient rights," are having profound and multifaceted impacts, particularly in regional healthcare settings. In rural and regional areas, these changes are even more pronounced due to the unique challenges these communities face, such as resource limitations, geographic isolation, and an aging population. The integration of AI has already begun to significantly influence areas like differential diagnosis, image interpretation, and other forms of information analysis, suggesting that the roles of healthcare professionals are poised to undergo substantial change in the AI era [24]. This transformation is especially critical in regional medicine, where healthcare workers often need to provide comprehensive care across a wide range of specialties, requiring a more flexible skill set and the ability to leverage AI tools to compensate for limited local expertise. Patients themselves are expected to play a more active role in managing their own health by using AI to acquire additional knowledge about their conditions and symptoms. As a result, healthcare professionals must continuously reflect on their roles in regional healthcare and rethink their approach to professionalism in the patient-physician relationship [25,26].

To adequately prepare healthcare professionals for these transformations brought about by AI, traditional medical education is no longer sufficient. There is a growing expectation that future healthcare providers will need to develop broader perspectives, not only in the clinical and technical aspects of medicine but also in humanistic and administrative areas. They will need to anticipate changes in their local communities and contribute to the development of healthcare personnel who are well-equipped to handle the unique challenges of regional healthcare. This is especially true in rural areas, where medical professionals often serve as the primary or sole healthcare providers and are required to address a wider range of medical issues than their urban counterparts. To support this, there is a call for the creation of new curricula that align with these evolving demands within specialized medical courses, particularly in regional medical education. Medical schools are expected to integrate participatory clinical training with initiatives that promote regional medical education, especially in the context of AI. Senior medical students, acting as "student doctors" during their clinical training, will increasingly have their medical practices made public [12], fostering transparency and trust within the community. At the same time, there is a growing emphasis on the need for students to acquire comprehensive clinical skills over the course of four years, with seamless integration of training during the fifth and sixth years of medical school and the early stages of clinical practice. This approach aims to ensure that medical graduates are fully prepared to respond to the needs of regional healthcare systems in a holistic manner, equipping them to manage the complexities of delivering care in rural and underserved areas [27]. In this context, all medical students, not just those enrolled in regional training programs, are expected to develop competencies in regional medical care that are aligned with the demands of the AI era [28].

**Figure 1:**
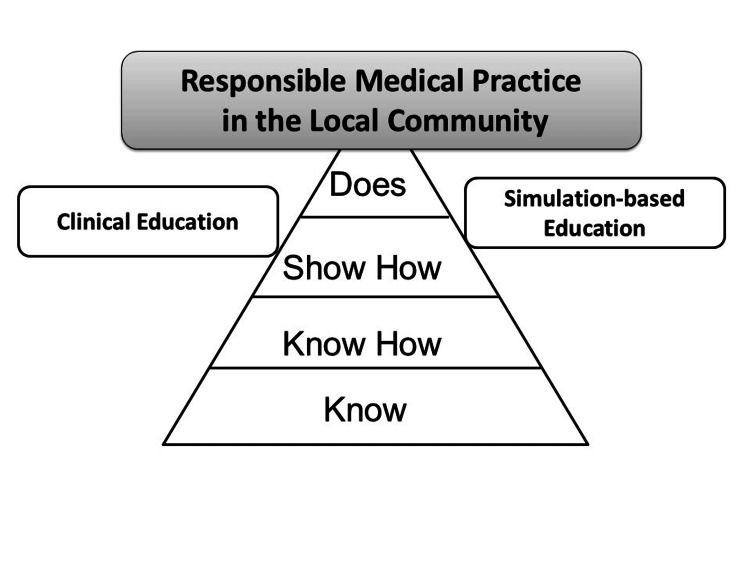
Millers’ pyramid for responsible medical practice in the local community Image Credits: Nobuyasu KOMASAWA

In considering how these competencies should be acquired, it is important to refer to frameworks like Miller’s Pyramid of Learning, which emphasizes the progression from theoretical knowledge to practical application. To better equip medical students for the future, regional medical education must be introduced earlier in their training, running in parallel with courses in mathematics, data science, and AI. This approach should begin in the theoretical and lower years of study, rather than waiting until the clinical training stage, to ensure that students develop the foundational skills necessary to navigate the complexities of modern medicine in the AI era (Figure 1) [29].

Educational content should include "systematic interprofessional education in the role of new healthcare providers in the AI era" and "career design education for the continuously evolving local community and regional healthcare" [30,31].

The need for "collaborative" education with a focus on interdisciplinary and regional collaboration

To ensure smooth interdisciplinary collaboration in the future of regional healthcare, medical universities have introduced courses on "interprofessional education," aiming to foster cooperation and teamwork among various healthcare professions [32]. This education is crucial because contributing effectively to regional healthcare, particularly in rural and underserved areas, requires not only expertise in fields such as medicine, nursing, and clinical psychology but also the ability to collaborate and communicate constructively with professionals from other disciplines. In regional healthcare settings, where resources are often limited and professionals must work closely to provide comprehensive care, successful delivery hinges not only on deep professional knowledge but also on maintaining a cooperative and open attitude toward other fields. This ensures that healthcare professionals, such as doctors, nurses, and welfare workers, can work together seamlessly to meet the unique challenges faced by regional populations, such as geographic isolation and higher rates of chronic illness [33,34]. To meet the demands of interdisciplinary collaboration in regional healthcare, it is essential to educate healthcare students and professionals on the roles and responsibilities of other disciplines, helping them develop communication skills that encourage mutual understanding and cooperation, particularly in the context of delivering care to diverse and often underserved populations [35].

However, a fundamental prerequisite for practicing effective interprofessional collaboration is establishing strong "intra-professional collaboration" and building "responsible professional competencies." In regional healthcare, where teams may consist of only a few practitioners who must cover a broad range of services, it is especially important that professionals first enhance their ability to work cohesively within their own discipline. This includes improving teamwork and communication skills within their own medical department or field, which is vital for rural areas where resources are stretched thin and healthcare professionals often need to take on multiple roles. Without the ability to function as an effective team member within one's own discipline, collaboration between different medical departments or professions is unlikely to succeed. The inability to collaborate within one's own profession creates a significant barrier to achieving interdisciplinary collaboration, especially in regional healthcare settings, where multiple professions need to work together seamlessly. In these regions, professionals from different disciplines, such as doctors, nurses, pharmacists, and social workers, must work in unison to provide holistic care to patients, many of whom require a combination of medical, welfare, and long-term care services. Moreover, within the regional comprehensive care system, where health, medical care, and welfare are intertwined, collaboration between diverse entities, including hospitals, clinics, and welfare organizations, is indispensable. This system is particularly vital in rural areas, where patients rely on a tightly integrated network of care providers. Therefore, there is a growing need for gradual, systematic "collaborative" education that begins with developing teamwork within one's profession and extends to collaboration within the broader regional comprehensive care system [34]. Simulation-based behavioral education may prove to be an effective tool in fostering such collaborative skills, as it allows professionals to practice real-life scenarios in a controlled, educational setting, enhancing their teamwork abilities across disciplines. This is particularly beneficial in rural healthcare, where practitioners must be prepared to handle a wide variety of cases with limited on-site resources [35].

As we enter an era increasingly shaped by AI-driven advancements, the roles and responsibilities of various healthcare professions are gradually evolving. The learning objectives in healthcare education curricula are expected to shift to reflect these changes. It is anticipated that the curriculum of each profession will need to be adjusted slightly to accommodate the new challenges posed by AI, including how different healthcare professionals interact with AI systems and how their roles may change over time [36]. Recognizing these changes and actively embracing interdisciplinary collaboration becomes more critical than ever, particularly in regional medicine, where healthcare workers are often required to perform tasks across a range of specialties. Beyond healthcare and welfare professionals, collaboration in regional healthcare is likely to expand to include administrative personnel and information managers, reflecting the growing need for a more integrated approach to healthcare. This expansion will help ensure sustainability in medical care, especially as it adapts to the evolving demands of an AI-driven society. In rural areas, where healthcare systems are more fragile and often rely heavily on local support structures, the integration of AI can help streamline processes, but it also requires a concerted effort in training professionals who can manage and work alongside AI systems while maintaining a strong human connection with patients.

In addition, interprofessional collaboration is expected to be applied across a broad spectrum of healthcare settings, from acute care to chronic care, and should encompass all stages of a patient’s life. This comprehensive approach will ensure that healthcare professionals are equipped to meet the needs of patients throughout their entire healthcare journey. The practical implementation of these collaborations will span across multiple stages, catering to both immediate, urgent medical needs and long-term, chronic care management. By fostering collaboration not just within healthcare but also across sectors such as social welfare, administration, and information management, the regional healthcare system will be better positioned to provide holistic and sustainable care for all its patients. This is particularly critical in rural and underserved areas, where care continuity and seamless collaboration between local hospitals, clinics, and welfare organizations are key to addressing both acute and chronic health issues effectively.

Possibility of simulation education support concurrent with regional participatory clinical training

All healthcare professionals, regardless of their specific roles, contribute significantly to treating and caring for patients and citizens living in rural and regional areas as long as they are engaged in patient care and medical treatment [37,38]. This is because regional healthcare operates as a key component of a comprehensive regional care system that integrates "healthcare," "medicine," and the "welfare system." In rural settings, where healthcare resources may be scarce and geographic isolation is a factor, this integrated system is particularly vital. Healthcare professionals work together to meet the diverse and often complex needs of the community, ensuring that patients receive holistic care that spans medical treatment, preventive healthcare, and social welfare support. This collaborative approach is essential for maintaining the health and well-being of local populations, many of whom may face barriers to accessing specialized care in urban centers.

When student doctors participate in regional healthcare through participatory clinical training, they are exposed to a wide range of medical situations that require a variety of skills. These skills are not limited to technical medical procedures but also include non-technical skills such as communication, teamwork, and decision-making, which are especially crucial in rural settings where healthcare professionals must often cover multiple roles and resources may be limited [39, 40]. To help student doctors acquire these necessary skills, concurrent simulation-based education is essential. In regional healthcare, where emergencies such as trauma or cardiac events may occur far from specialized centers, it is critical that student doctors are prepared for high-invasiveness medical procedures. Simulation education should be designed to support and complement the hands-on experience gained during regional participatory clinical training. In particular, there is a need for simulation education that focuses on high-invasiveness emergency procedures, which aligns with the specific learning goals of students engaged in rural clinical training [41,42]. For example, during their regional clinical placements, student doctors may encounter situations where they need to perform advanced emergency medical procedures, such as trauma care or advanced cardiac life support [43,44]. In these cases, they can first develop their basic skills through simulation training at a university simulation center before applying those skills in real-world, resource-limited settings [45]. For example, Jichi Medical University has been accepting medical students since 1972 for medical care in rural areas in Japan. The Jichi Medical University's Simulation Education Center has played an essential role in basic medical skill training [46]. Establishing a high-quality simulation training environment is crucial for ensuring that student doctors are well-prepared and able to practice medicine responsibly and safely within local communities, where healthcare infrastructure may be more limited than in urban areas.

As we enter the AI-driven era, where society is increasingly information-based, the regional healthcare system has undergone significant changes. These changes have introduced new educational formats, including the use of information and communication technology (ICT) and remote learning methods, which are becoming part of the new normal in medical education [26]. These technological advancements are particularly beneficial in rural healthcare, where ICT can help overcome geographic barriers by providing access to specialized knowledge and support. Despite the widespread adoption of these new technologies and educational media, including advanced simulators, the fundamental principles of medical education remain unchanged. The core of medical education still revolves around experiential learning, where students learn best by doing and reflecting on their experiences. In rural settings, where student doctors may have the opportunity to engage more directly with a wide range of medical issues, hands-on learning becomes even more critical. Thus, while AI and ICT can enhance access to information and provide new learning tools, the foundation of medical education remains experiential, particularly in the context of regional healthcare, where practical skills and adaptability are paramount [47,48].

Therefore, even as the AI-driven society progresses, simulation-based medical education, grounded in experiential learning theory, remains crucial for helping students acquire foundational clinical skills, especially in rural and regional healthcare contexts [48,49]. This hands-on approach ensures that future healthcare professionals are well-equipped to handle the complexities of patient care in rural settings while also integrating new technologies and approaches into their practice. The integration of simulation-based education into regional participatory clinical training plays a vital role in preparing student doctors for the diverse challenges they will face in rural and regional healthcare settings. By combining hands-on clinical experience with targeted simulation training, students can develop both the technical and non-technical skills needed for responsible, high-quality medical practice in underserved areas. As the regional healthcare system continues to evolve in response to advancements in AI and information technology, simulation education will remain a key tool in ensuring that medical students are well-prepared to meet the unique needs of their communities and provide sustainable, holistic care [50].

## Conclusions

Regional healthcare education aims to provide accessible, community-centered medical services, respond to the specific healthcare needs of different regions, and ensure that medical care is fairly distributed, contributing to the well-being and sustainability of local communities. Yet, significant challenges remain, such as the disparity between urban and rural healthcare resources, the low retention of physicians in rural areas, and the necessity for region-specific educational programs that address local health concerns. To tackle these issues, enhancing the quality of regional medical education is crucial. This can be done by improving the clinical training environment, offering attractive incentives to healthcare professionals working in rural areas, and incorporating modern educational techniques like simulation-based learning. Additionally, updating medical education to meet the demands of a data-driven healthcare environment and encouraging interdisciplinary collaboration is key to equipping healthcare workers to address the evolving needs of regional healthcare. By addressing these challenges, we can create a more robust and fair healthcare system, which not only improves the well-being of rural populations but also strengthens the healthcare landscape of the entire country.
